# The Effect of Voltage Stabilizers on the Electrical Resistance Properties of EPDM Bulk for Cable Accessories

**DOI:** 10.3390/polym17182523

**Published:** 2025-09-18

**Authors:** Zhongyuan Li, Zhen Zhang, Chang Liu, Chenyang Ma, Xueting Wang

**Affiliations:** Electric Power Research Institute, State Grid Heilongjiang Electric Power Co. Ltd., Harbin 150030, China; 2220310150@stu.hrbust.edu.cn (Z.Z.); 2220310178@stu.hrbust.edu.cn (C.L.); 2220300046@stu.hrbust.edu.cn (C.M.); 2020310226@stu.hrbust.edu.cn (X.W.)

**Keywords:** EPDM, voltage stabilizer, DC breakdown field strength, surface breakdown

## Abstract

As a critical component in high-voltage cable accessories, ethylene-propylene-diene monomer (EPDM) reinforced insulation faces severe issues of surface discharge and bulk breakdown at the insulation interface. To enhance the electrical resistance of EPDM bulk and insulation interfaces, the 4-allyloxy-2-hydroxybenzophenone was employed as a voltage stabilizer to modify EPDM. A systematic study was conducted on the influence of the voltage stabilizer on the DC breakdown strength of EPDM, the anti-migration properties of the voltage stabilizer, and its effect on the surface breakdown voltage of EPDM. Additionally, pressure and surface breakdown test setups were designed. The results indicate that the DC breakdown strength of EPDM decreases with increasing external pressure, and this decline is more pronounced in EPDM modified with the voltage stabilizer. Surface breakdown experiments demonstrate that the voltage stabilizer has a positive effect on improving the surface breakdown voltage of EPDM, with a more significant enhancement observed at the EPDM/XLPE bilayer dielectric interface. Surface potential tests reveal that the grafted voltage stabilizer introduces numerous shallow traps, inhibiting surface charge accumulation and thereby increasing the surface breakdown voltage.

## 1. Introduction

Ethylene Propylene Diene Monomer (EPDM) rubber offers advantages such as low loss, resistance to partial discharge, and UV protection. EPDM is widely used in insulation for mining cables, nuclear power plant cables, and marine cables, among others. Its service life is approximately ten times that of other rubbers. As a result, EPDM is more extensively applied in high-voltage cables and accessories both domestically and internationally [1,2,3].

However, under actual operating conditions, uneven electric field distribution can occur, leading to localized electric field concentration and partial discharge, ultimately affecting the stable operation of EPDM rubber cables and accessories. If the EPDM cable joint is not tightly connected or the insulation is exposed, discharge is prone to occur at this point. The discharge gradually forms leakage traces, leading to the degradation of insulation performance and even eventual failure. The surface discharge occurring at the cable joint is a discharge issue at the interface between solid and gas. Under the influence of the electric field, the distribution of the electric field at the gas–solid interface is uneven, making it prone to surface discharge. Surface discharge will create discharge traces on the insulating interface, reducing the surface conductivity of the insulation. In severe cases, it may lead to breakdown along the insulating surface, causing permanent damage to the insulation [4,5,6].

The scholars have conducted extensive research on surface discharge of insulating materials, with the core issue focusing on exploring the surface charge accumulation mechanism of composite materials and suppressing the occurrence of surface discharge by regulating the distribution of surface charges. Studies have shown that doping nanoparticles can modify the trap energy levels of polymers, influence the charge transport within the material, and thereby enhance the surface breakdown field strength of the polymer. For instance, Wang et al. doped epoxy resin with nano-ZnO, introducing shallow traps that accelerated surface charge dissipation and improved the surface withstand voltage performance of the composite material [7]. Li Shengtao et al. modified nano-TiO_2_ particles with a silane coupling agent and doped 1.0 wt% TiO_2_ into epoxy resin or low-density polyethylene [8], finding that nanoparticle doping introduced deep traps, thereby suppressing surface charge accumulation and increasing the surface flashover voltage of the insulator. It is evident that scholars have conducted considerable research on discharge behavior at the interfaces of different insulating materials, with modification methods primarily focused on nanoparticle doping. However, nanoparticles are prone to agglomeration, making them difficult for engineering applications.

The internal structure of cable accessories, due to the presence of composite insulation interfaces and concentrated electric field stress, has become a weak link in high-voltage cable insulation and a typical location for operational failures [9]. Operational failure cases of cable accessories have shown that surface discharge in the composite insulation majorly accounts for operational failures in cross-linked polyethylene (XLPE) power cables and cable accessories, whilst the pressure on composite insulation interface is the dominant factor in generating surface discharge [10]. Excessive pressure on the composite interface between elastomer reinforce insulation and XLPE main insulation in cable accessories can lead to difficulties in installing cable accessories and even the “bamboo joint” phenomenon in cable insulation during load operation [11]. Recent studies have demonstrated that the pressure at reinforce/main insulation interface (grip force) is generally controlled in the range of 0.1–0.25 MPa to meet the electrical strength requirements without causing installation difficulties or cable insulation damage [12]. Appropriate interface pressure of the accessory insulation is only a sufficient condition for achieving high electrical strength at the interface; the interface electrical strength also depends on the rational structural design of the accessory and the correct installation process [13]. Moreover, the effect of the pressure on the composite insulation interface in cable accessories on the accessory insulation strength also changes with variations in interface roughness, interface coating conditions, cable temperature rise, and accessory insulation stress relaxation [14].

Currently, engineering approaches to improve the interfacial insulation strength of cable accessories mainly include increasing interfacial pressure and coating with insulating silicone grease [15,16]. However, existing research indicates that excessive interfacial pressure can lead to permanent compression deformation of the XLPE insulation layer after cable load changes. Moreover, excessive interfacial pressure can cause microcracks in rubber materials under long-term stress, posing a threat to the operation of cable accessories. Additionally, while coating the interface with silicone grease can temporarily improve surface breakdown strength, the swelling effect of the grease not only reduces the mechanical and electrical performance of the rubber interface [17,18]. Therefore, there remains a lack of effective material modification methods to improve the electrical resistance of rubber-reinforced insulation bulk and interfaces.

Polar molecular grafting modification technology has become a research hotspot in recent years due to its low additive content and excellent improvement of polymer insulation performance. Voltage stabilizers, a special type of polar molecule, are commonly used as additives to enhance the electrical tree resistance of PE or XLPE. Recent studies have further demonstrated that grafting polar-group molecules (e.g., CAAE or MAH) onto XLPE chains can introduce deep charge traps (~1.1–1.2 eV), suppress charge injection and carrier transport, and raise AC breakdown strength by >20% at temperatures up to 150 °C [19]. Equally, surface-grafted AOHBP voltage stabilizer on EPDM was reported to create a high-density trap layer that significantly delays electrical-tree initiation and growth under DC stress [20]. These findings corroborate that covalently bonded polar stabilizers outperform conventional physical blending in both dielectric strength and long-term aging resistance. However, no researchers have yet attempted to apply them to improve the electrical resistance of reinforced insulation materials in cable accessories. Currently, voltage stabilizers are only used in the insulation bulk, and their potential to enhance the electrical resistance of insulation interfaces remains unknown. If the mechanism of voltage stabilizers can be effectively utilized and introduced into EPDM to improve the electrical resistance of reinforced insulation bulk, it would significantly reduce the difficulty of insulation structure design for high-voltage cable accessories, improve their long-term operational reliability, and further increase their working voltage levels.

Therefore, the present study attempts to use voltage stabilizers as additives for EPDM, combining experiments and theoretical calculations to analyze their impact on the surface breakdown characteristics of EPDM, providing a new direction for the research of novel insulating materials. Combining chemical modification schemes and charge trap regulation theory, the present study explores ameliorating the interfacial insulation performance of EPDM material used for cable accessory by uniformly grafting a preferable species of voltage stabilizer (with an optimized grafting content of 0.5 phr), which avoids the agglomeration problem in nanodielectrics. In particular, the dual evolution law of density attenuation—energy level shift for the shallow charge traps introduced by grafting voltage stabilizer grafting under both pressure and electric field is revealed. A quantitative mechanism correlating pressure, charge trap and surface breakdown is proposed, filling the research gap in molecular-level charge trapping mechanism under pressure environment. Targeting the practical application scenarios of cable accessories, the voltage stabilizer modification for the EPDM/XLPE bilayer dielectric interface is pioneered, finding a pressure threshold for interface insulation design in cable accessory, which breaks through the current research limitations that predominantly focus on the bulk properties of single dielectrics.

## 2. Sample Preparation and Testing

### 2.1. Material Preparation

**Figure 1:**
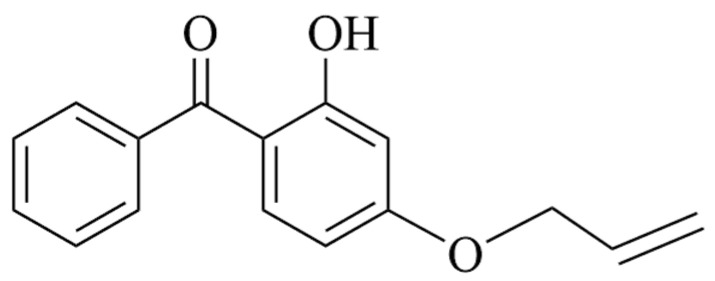
Molecular structure of AOHBP voltage stabilizer.

Recent high-impact literature related to cable accessory insulation has highly suggested two important voltage stabilizers, acrydoxy-phenylacetone and 4-acrylicoxy-2-hydroxydiphenone (VPE and AOHBP) for improving insulation performance of EPDM material by chemical grafting, which demonstrates AOHBP (containing dual hydroxyl and benzophenone functional groups) with a grafting content of 0.5 phr is the most preferred than VPE (containing single-functional-group) and other densities of grafting modification [21,22]. Therefore, grafting 0.5 phr AOHBP is selected and compared with 1.0 phr as the chemical modification schemes on EPDM material in the present study. The raw materials used in this paper include ethylene propylene diene particles (4725P, Dow Chemical Company, Midland, MI, USA) as monomers and 4-propenyloxy-2-hydroxybenzophenone (AOHBP, 98% purity, Sinopharm Chemical Reagent Co., Ltd., Shanghai, China) as voltage stabilizer modification agent being shown for the molecular structure in Figure 1.

The modified EPDM material was prepared using melt blending and hot pressing. Melt blending: First, EPDM particles were placed into a torque rheometer at 110 °C with a rotational speed of 60 rpm for mixing until the particles were completely melted. Then, 0.5 or 1.0 phr voltage stabilizer and 2.0 phr DCP were added and mixed for 8 min to obtain the EPDM mixture.

Hot pressing: First, the mixture was placed into a 100 mm × 100 mm × 3 mm mold and preheated in a flat vulcanizing machine at 110 °C for 5 min, followed by pressing at 15 MPa for 15 min to form the material. Next, the hot-pressed sample was vulcanized in the flat vulcanizing machine at 175 °C and 15 MPa for 30 min. Finally, it was cooled under 15 MPa pressure. The obtained sample was then placed in an 80 °C vacuum drying oven for degassing before testing.

### 2.2. Infrared Spectrum

To analyze the effect of the voltage stabilizer on the molecular structure of EPDM, Fourier-transform infrared spectroscopy (FTIR) was used to characterize the modified EPDM material. The FTIR test range was 500–4000 cm^−1^ with a resolution of 2 cm^−1^, and the sample thickness was 150 μm. During testing, it was observed that when small amounts of voltage stabilizer were added, the characteristic absorption peaks of double bonds and related structures in the infrared absorption spectrum were weak, making it difficult to compare changes before and after crosslinking. Therefore, the infrared absorption spectra of modified materials with 3.6 phr of voltage stabilizer were tested separately to analyze the changes before and after crosslinking, thereby investigating possible chemical reactions of the voltage stabilizer molecules during the EPDM crosslinking process.

### 2.3. Crystallization Characteristics

A Mettler Toledo DSC822e differential scanning calorimeter (DSC) Manufactured by METTLER TOLEDO, Switzerland was used to test the crystallization and melting processes of EPDM and AOHBP/EPDM materials. The procedure was as follows: First, the test samples were cut into small pieces, and approximately 5–10 mg of the sample was weighed using a high-precision electronic balance and placed in a crucible. To ensure contact with the atmosphere, a small hole was punctured in the center of the crucible lid, which was then sealed and pressed tightly using a compression device. The crucibles containing the test sample and reference sample were placed into the sample chamber, and the programmed temperature was set for testing.

The DSC test was conducted under a high-purity nitrogen flow rate of 50 mL/min. The temperature control scheme was set as follows: First, the temperature was increased from 25 °C to 150 °C at a rate of 10 °C/min and held at 150 °C for 5 min to eliminate the influence of thermal history on the test results. Subsequently, the temperature was decreased from 150 °C to 25 °C at 10 °C/min and held at 25 °C for 5 min. Finally, the temperature was increased again from 25 °C to 150 °C at 10 °C/min and held at 150 °C for 5 min.

### 2.4. Surface Flashover

**Figure 2:**
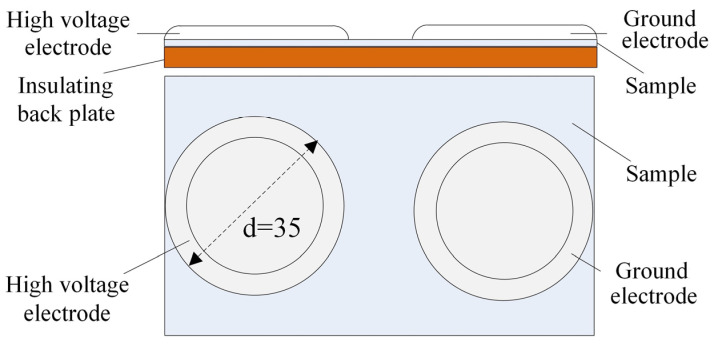
Test device for surface breakdown.

A surface flashover test setup was used, as shown in Figure 2. Two cylindrical electrodes with a diameter of 35 mm served as the high-voltage electrode and ground electrode, respectively, with an electrode gap of 4 mm. To prevent gas discharge between the electrodes, the edges were rounded with a 5 mm radius. The electrode surfaces were ensured to have good contact with the EPDM sample. The power supply was turned on, and the voltage was gradually increased at a rate of 0.3 kV/s until discharge occurred between the electrodes, resulting in flashover. The voltage at this point was recorded as the surface flashover voltage.

### 2.5. Pressure Breakdown Device

**Figure 3:**
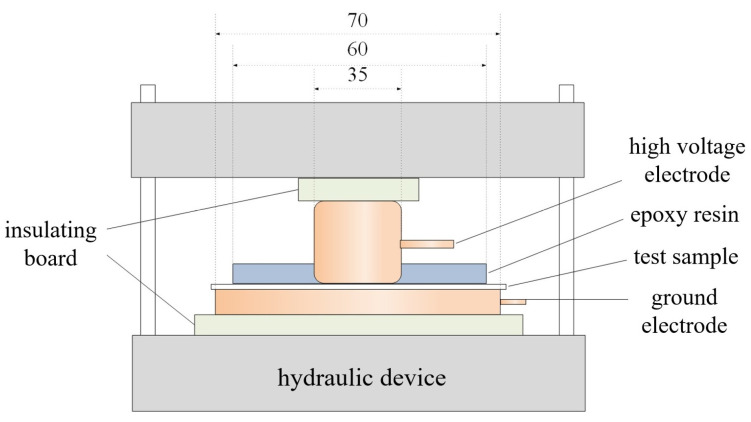
Test device for breakdown strength under pressure.

The pressure breakdown test setup used is illustrated in Figure 3, comprising a hydraulic device, a high-voltage electrode encapsulated in epoxy resin, a grounding electrode, and an insulating plate. The hydraulic device provides stable and uniform pressure, with the applied pressure displayed in real-time via a pressure gauge. To prevent discharge from the electrodes to the hydraulic device, a circular insulating plate with a diameter larger than that of the electrodes is employed to isolate them. Cylindrical electrodes are used for the breakdown strength test, with diameters of 35 mm for the high-voltage electrode and 70 mm for the grounding electrode. To avoid partial discharge, both electrodes feature rounded edges with a radius of 5 mm. Additionally, to ensure uniform pressure distribution at the contact between the sample and electrode edges, the lower end of the high-voltage electrode is encapsulated with a 7 mm-thick, 60 mm-diameter epoxy resin. A thin layer of conductive silicone grease is applied to the electrode surfaces in contact with the sample during testing to maximize pressure uniformity.

During the test, a specific pressure is uniformly applied to the sample. The DC power supply is then activated, and the voltage is increased at a rate of 0.3 kV/s until the sample breaks down. The large current generated at the moment of breakdown is detected by the operating box, which automatically disconnects the circuit. The voltage at this instant is recorded as the breakdown voltage. Given the inherent dispersion in breakdown field strength data, 15–20 samples of each material are tested to accurately determine the DC breakdown field strength pattern. The two-parameter Weibull distribution is applied to statistically analyze the DC breakdown field strength of the samples under pressure.

### 2.6. Double-Layer Dielectric Breakdown Test Device

**Figure 4:**
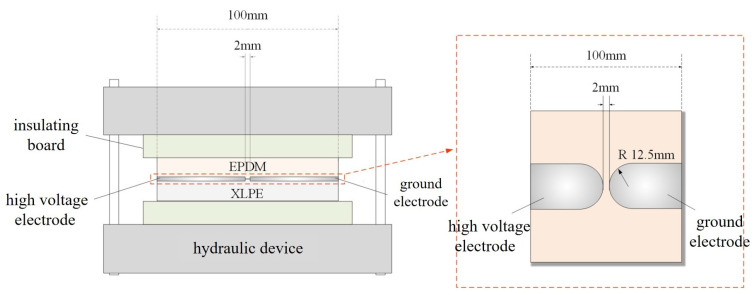
The setup diagram for breakdown voltage of Double-layer dielectric interface.

The double-layer dielectric interface breakdown voltage test device is shown in Figure 4, consisting of a hydraulic device, insulating plates, a double-layer dielectric sample, a high-voltage electrode, and a grounding electrode. The hydraulic device is used to provide stable and accurate pressure, which can be displayed in real-time by a pressure gauge. The insulating plates separate the hydraulic device from the sample to prevent discharge. The prepared double-layer dielectric sample is placed between the two insulating plates, and a pressure of 0.3 MPa is applied to the sample via the hydraulic device. A DC voltage is then applied to the interface electrode at a rate of 0.3 kV/s. When the sample breaks down, the voltage at that moment is recorded as the breakdown voltage of the double-layer dielectric interface.

### 2.7. Surface Potential

**Figure 5:**
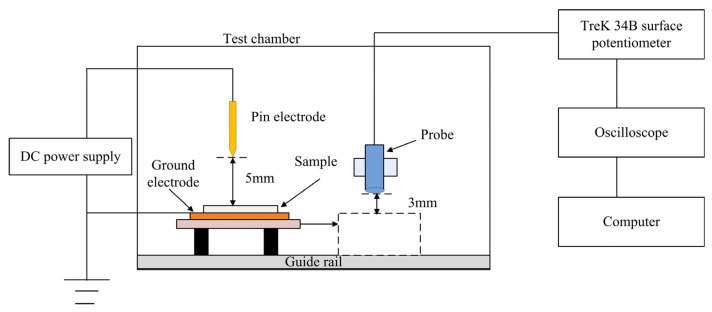
Schematics of testing equipment with ISPD method.

The Isothermal Surface Potential Decay Method (ISPD) can characterize trap energy level distribution and charge transport processes. The film sample is charged using a corona discharge method, with the experimental measurement system illustrated in Figure 5. The needle tip is positioned 5 mm from the grid, and the grid is 5 mm from the sample surface. The needle electrode and grid are connected to a high-voltage source, while the back electrode, made of aluminum, is grounded during measurement. The potentiometer used is a Trek 341B (Made in the USA by Trek, Inc., New York, NY, USA), with the electrostatic probe fixed on a sliding rail bracket approximately 3 mm from the sample surface. In this study, negative corona charging was selected, so the needle electrode and grid were charged at −8 kV and −5 kV, respectively, for 5 min. The sample was then moved under the electrostatic probe for measurement, with a testing duration of 20 min, conducted at room temperature.

For samples with thicknesses ranging from micrometers to millimeters, the calculated trap energy level distribution can be approximately regarded as the bulk trap energy level distribution [23,24]. The double exponential function can effectively fit the surface potential decay curve over time, with the fitting formula as follows:

(1) $$U=Ae^{\frac{-t}{\tau_{1}}}+Be^{\frac{-t}{\tau_{2}}}$$

where *A*, *τ*_1_, *B*, and *τ*_2_ are fitting parameters. According to the theory proposed by Simmons and Tam, assuming that during the surface potential decay process, charges detrap without subsequent retrapping and ignoring the recombination behavior of holes and electrons, the trap distribution in the sample can be calculated based on the surface potential decay curve:

(2) $$E_{t}=kT\ln(\nu t),\;N(E)=\frac{4\varepsilon_{0}\varepsilon_{1}}{qkTd^{2}}\left|t\frac{dU}{dt}\right|,\;N(E)=\frac{4\varepsilon_{0}\varepsilon_{1}}{qkTd^{2}}\left|t\frac{dU}{dt}\right|$$

where *E*_t_ represents the trap energy level, *N*(*E*) is the trap density, *d* is the sample thickness, *ε*_0_ and *ε*_1_ are the vacuum permittivity and relative permittivity, respectively, *ν* is the electron escape frequency, *q* is the electron charge, and *k* is the Boltzmann constant.

## 3. Results and Analysis

### 3.1. Infrared Characterization of Voltage Stabilizer-Modified EPDM

**Figure 6:**
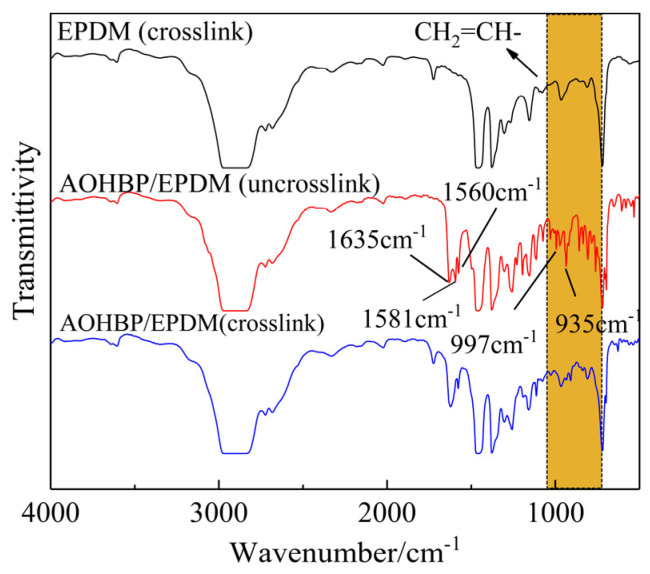
Infrared transmission spectra of AOHBP/EPDM (0.5 phr grafting content) before and after crosslinking process in comparison to neat EPDM. The orange area in the figure is caused by the out-of-plane wagging vibration of the C-H bond in CH2=CH-.

Figure 6 shows the molecular structure characterizations of voltage stabilizer-modified EPDM (AOHBP/EPDM) with a grafting content of 0.5 phr before and after crosslinking process in comparison to neat EPDM. It can be observed that the broad absorption peak at 2800–3000 cm^−1^ is caused by the C-H stretching vibration of the -CH_2_- segments in the EPDM matrix. The absorption peak between 1430 cm^−1^ and 1490 cm^−1^ arises from the in-plane bending vibration of C-H in -CH_3_. The absorption peaks at 720 cm^−1^ and 962 cm^−1^ are attributed to the out-of-plane bending vibration of C-H in -CH=CH-, while the peak at 1723 cm^−1^ corresponds to the absorption of the C=O group in linear saturated ketones. All samples were vacuum-treated prior to testing.

In Figure 6, the characteristic peaks of the uncrosslinked AOHBP/EPDM sample at 1635 cm^−1^, 1560 cm^−1^, and 1581 cm^−1^ correspond to the C=O absorption peak in the AOHBP molecule, the benzene ring vibration absorption peak, and the vinyl C=C double bond vibration peak, respectively, while the peaks at 997 cm^−1^ and 935 cm^−1^ are caused by the out-of-plane wagging vibration of the C-H bond in CH_2_=CH- of AOHBP. After crosslinking, the characteristic peaks at 997 cm^−1^, 935 cm^−1^, and 1581 cm^−1^ almost disappear, while the other characteristic peaks remain. This indicates that the vinyl groups in AOHBP are largely consumed during the crosslinking process, demonstrating that AOHBP and VPE can be grafted onto the EPDM molecular chains through their vinyl addition reaction.

### 3.2. Melting and Crystallization Characteristics of Voltage Stabilizer-Modified EPDM

**Figure 7:**
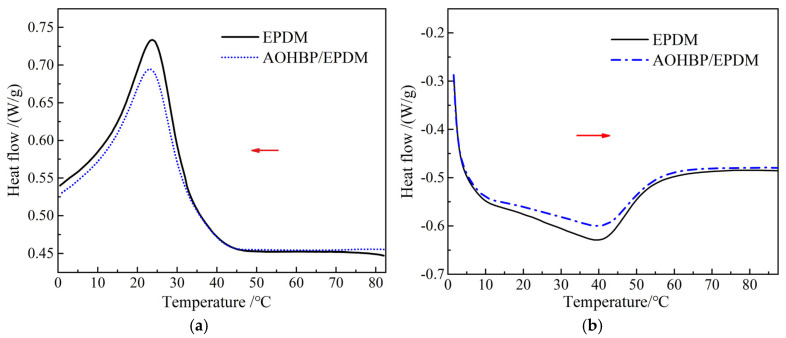
DSC heat flow spectra in (**a**) crystallization and (**b**) melting processes of EPDM and 0.5 phr AOHBP/EPDM. Among them, the red arrow in (**a**) indicates the cooling direction, while the red arrow in (**b**) indicates the heating direction.

Based on the analysis in Section 3.1, it is concluded that voltage stabilizer molecules can be grafted onto the EPDM macromolecules. During the grafting process, the regularity of the macromolecular structure is inevitably altered, thereby affecting the crystallization characteristics of the material. Considering practical applications, further research was conducted to investigate the impact of voltage stabilizer grafting on the crystallization performance of EPDM. The DSC test results of EPDM and AOHBP/EPDM (0.5 phr grafting content) are shown in Figure 7, and the crystallization parameters of the DSC curves are listed in Table 1.

The small molecule additives incorporated into polymers may inhibit crystallization by disrupting the regular arrangement of macromolecules or act as nucleating agents to promote heterogeneous nucleation. The crystallization peak temperature of EPDM and AOHBP/EPDM is around 23 °C, while the melting peak temperature is approximately 40 °C. The addition of voltage stabilizers to EPDM slightly lowers the crystallization peak temperature and slightly raises the melting peak temperature, but overall, the changes in these temperatures are not significant. Furthermore, as seen from the DSC curves in Figure 6, the peak areas of the crystallization and melting peaks decrease noticeably after adding voltage stabilizers to EPDM, indicating that the voltage stabilizers exert a certain inhibitory effect on the crystallization process of EPDM molecules.

**Figure 8:**
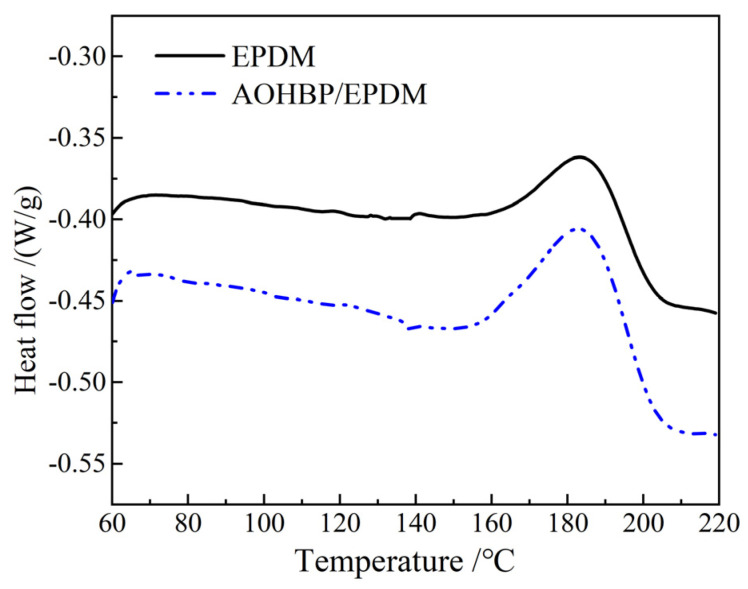
DSC curves in the crosslinking reaction processes of EPDM and 0.5 phr AOHBP/EPDM.

This section also employs differential scanning calorimetry (DSC) to investigate the heat flow changes during the crosslinking reaction of EPDM and 0.5 phr AOHBP/EPDM under a constant heating rate. The entire testing process was conducted under the protection of high-purity nitrogen with a flow rate of 50 mL/min. The programmed temperature was set to increase from 30 °C to 220 °C at a heating rate of 10 °C/min, followed by a 5 min isothermal hold, while recording the heat flow changes in the samples during heating. Since the crosslinking reaction releases heat, this section focuses solely on analyzing the exothermic peaks during the crosslinking process. The heat flow changes in EPDM and AOHBP/EPDM during the crosslinking reaction are illustrated in Figure 8, and the relevant parameters of the DSC curves during the crosslinking reaction are listed in Table 2.

The enthalpy change Δ*H* represents the total heat released per unit mass of material during the crosslinking reaction and can be used to compare the extent of the reaction. A larger Δ*H* indicates a more intense crosslinking reaction, as represented by the area of the exothermic peak in the graph. During the crosslinking reaction, the exothermic peak temperature of the materials remains nearly consistent at around 180 °C. As shown in Table 2, the reaction enthalpy change of the modified materials with voltage stabilizers is significantly greater than that of EPDM, further confirming that the voltage stabilizer molecules participate in the EPDM crosslinking reaction, leading to a more intense reaction process and a larger total reaction quantity in the modified materials.

### 3.3. Surface Breakdown Characteristics of AOHBP/EPDM

**Figure 9:**
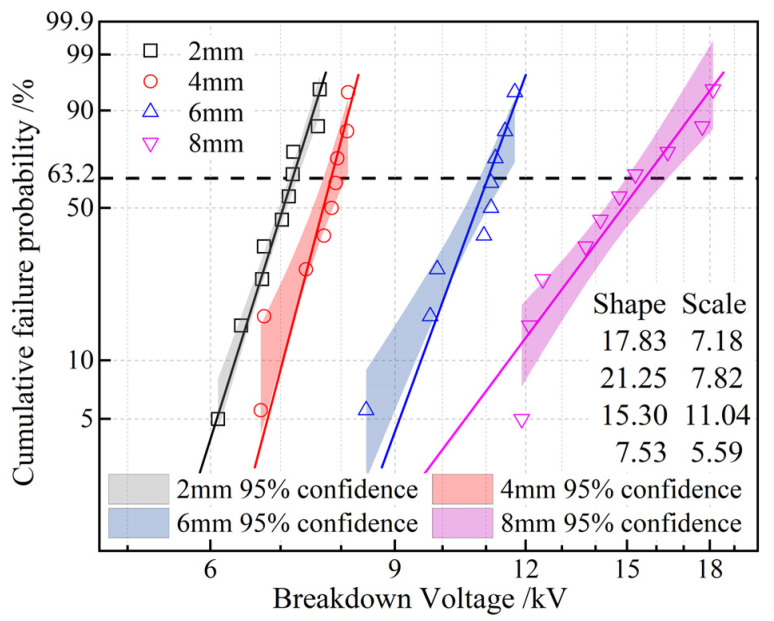
Two-parameter Weibull statistics on surface breakdown voltages of EPDM at various electrode distances.

It is generally believed that the breakdown voltage at the gas–solid or gas–liquid interface is lower than that of the solid itself. This is primarily due to differences in dielectric constant or conductivity between the two media at the interface, resulting in uneven electric field distribution and thus reducing the breakdown voltage at the interface. The Weibull distribution of the surface breakdown voltages of EPDM under various electrode distances ranging from 2 mm to 8 mm is shown in Figure 9. It can be observed that as the spacing increases, the surface breakdown voltage of EPDM gradually rises. This is because, in the early stages of discharge, ions slowly move from the high-voltage electrode to the ground electrode. When the voltage is sufficiently high, the ions gain enough energy to collide with free electrons on the EPDM surface, causing ionization and forming a uniform plasma layer near the high-voltage electrode. As the applied voltage further increases, the plasma layer moves toward the ground electrode, leading to breakdown. A larger spacing requires higher energy for the plasma layer to reach the ground electrode, resulting in a higher breakdown voltage. Additionally, the figure shows that when the electrode spacing is 4 mm, the breakdown voltage has a larger shape parameter and the best dispersion, so the electrode spacing is set to 4 mm for subsequent tests.

**Figure 10:**
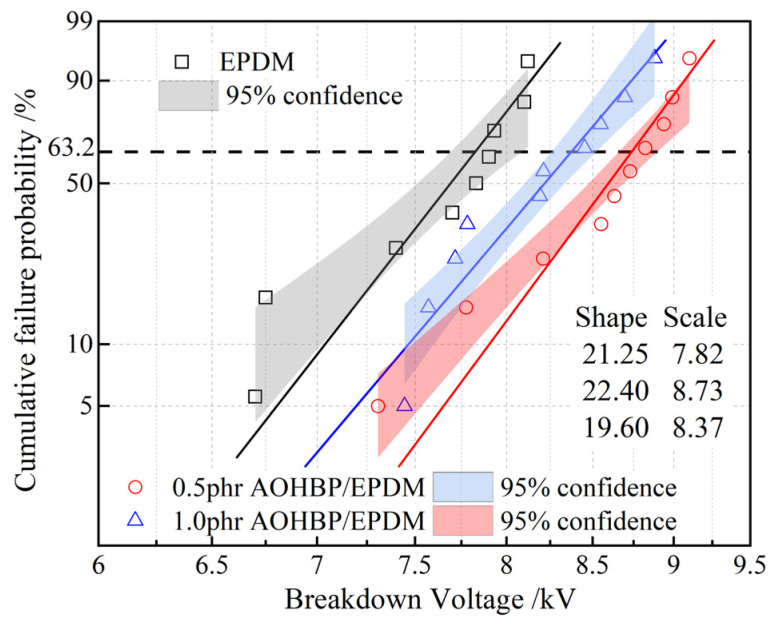
Two-parameter Weibull statistics on surface breakdown voltages of 0.5 phr and 1.0 phr AOHBP/EPDM compared with neat EPDM.

The Weibull distribution of the surface breakdown voltages of EPDM and AOHBP/EPDM with 0.5 phr and 1.0 phr grafting contents under a DC electric field is shown in Figure 10. It can be seen that adding voltage stabilizers improves the surface breakdown voltage of EPDM. Among them, the addition of 0.5 phr AOHBP enhances the breakdown voltage more effectively than 1.0 phr AOHBP, so 0.5 phr AOHBP/EPDM is selected for further study. The surface breakdown voltage of 0.5 phr AOHBP/EPDM reaches 8.73 kV, achieving 11.6% improvement over 7.82 kV of neat EPDM, more prominent than the relevant reports in reference [25] where the grafting voltage stabilizer improves EPDM surface breakdown voltage by 9.8%. The AOHBP molecule employed in this study features hydroxyl and benzophenone groups, exhibiting stronger charge-trapping capability compared to the single-functional-group stabilizer used in prior works.

**Figure 11:**
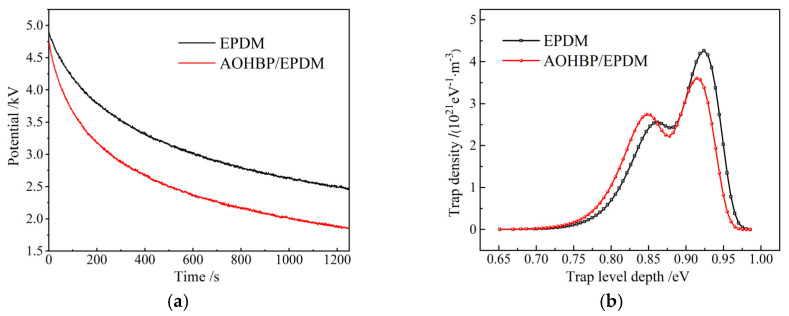
(**a**) Surface potential decays and (**b**) charge trap energy distributions of EPDM and 0.5 phr AOHBP/EPDM.

Surface breakdown in insulating materials is related to surface charges, and surface charge density is approximately proportional to the surface potential. Therefore, this study represents the dissipation of surface charges through surface potential decay characteristics, as shown in Figure 11. Figure 11a shows that under the same charging conditions, the initial potential of EPDM with voltage stabilizers is lower than that of neat EPDM, indicating that voltage stabilizers reduce surface charge accumulation. Moreover, compared to neat EPDM, the decay rate of EPDM with voltage stabilizers is significantly higher, facilitating surface charge dissipation. Figure 11b reveals that the deep trap center of neat EPDM reaches 0.92 eV, while the shallow trap center is 0.85 eV. After grafting AOHBP, the charge traps of EPDM becomes shallower, while the deep trap and shallow trap decreases and increases in density, respectively, both of which can release the kinetic energy of charge carriers and thus restrain them from gaining sufficient energy to cause collision ionization, effectively inhibiting surface breakdown in EPDM. ISPD measurements show AOHBP/EPDM’s shallow trap center at 0.83 eV with 42% higher density than neat EPDM, which supports the conclusion of shallow traps inhibiting charge accumulation as reported in reference [22] and highly suggests that shallow trap density strongly correlates with surface breakdown voltage, extending the literature-reported “trap density-dielectric strength” relationship.

### 3.4. DC Breakdown Characteristics Under Different Pressures

The interference fit between cable accessories and the cable body generates interfacial pressure, which directly affects the breakdown strength of EPDM [24]. If the interfacial pressure is too low, gaps may form at the interface. Due to the difference in relative permittivity between gas and solid, the electric field distribution at the gas–solid interface becomes uneven under an electric field, making surface discharge more likely. Discharge marks on the interface reduce the surface conductivity of the insulation and, in severe cases, lead to surface breakdown. Conversely, excessive interfacial pressure can cause difficulties in installing cable accessories and stress relaxation during operation [26].

**Figure 12:**
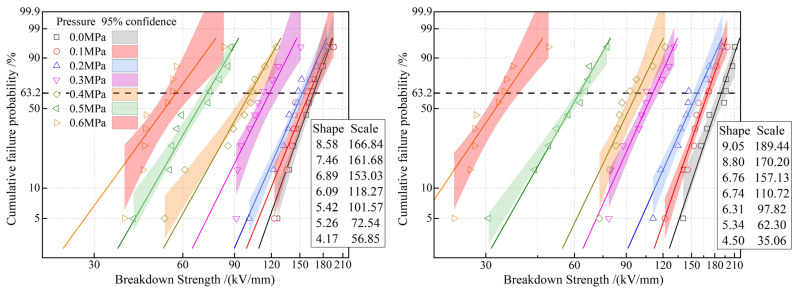
Weibull statistical dielectric breakdown strengths of EPDM (**left panel**) and 0.5 phr AOHBP/EPDM (**right panel**).

In the absence of external mechanical forces, the molecular chains of EPDM exhibit a relatively loose, random coil structure [27]. However, when subjected to external mechanical pressure, the interactions between the molecular chains undergo significant changes. Specifically, mechanical pressure reduces the distance between the molecular chains, thereby increasing the intermolecular forces [28]. Our experimental results show that within the mechanical pressure range of 0.1–0.6 MPa, the dielectric breakdown strength of EPDM decreases significantly with increasing pressure as shown in Figure 12. The changes in the molecular chain structure in EPDM caused by mechanical stress and the concentration of the electric field are the main reasons for the decrease in dielectric breakdown strength. From a microscopic perspective, the dielectric breakdown strength of EPDM is closely related to the structure and movement of its molecular chains. Under normal conditions, the random coil structure of EPDM molecular chains can effectively disperse the electric field, thereby enhancing the material’s dielectric breakdown strength [29]. However, when subjected to mechanical pressure, the distance between the molecular chains decreases, and the movement of the molecular chains is restricted, causing the electric field to concentrate in certain areas and leading to an overall decrease in dielectric breakdown strength [30]. Moreover, mechanical pressure can also cause minor deformations in the chemical bonds between the EPDM molecular chains. Although these deformations may not be apparent at the macroscopic level, they can alter the electron cloud distribution of the molecular chains at the microscopic level, thereby reducing the dielectric breakdown strength of EPDM materials [31]. In summary, when EPDM is subjected to external mechanical pressure, its molecular chain structure undergoes significant changes, leading to electric field concentration and restricted molecular chain movement, which results in a decrease in the dielectric breakdown strength of EPDM. Therefore, in practical applications, it is necessary to fully consider the impact of mechanical pressure on the electrical insulation properties of EPDM to ensure the reliability and safety of the material under specific conditions.

More important, when the pressure exceeds 0.3 MPa, the characteristic breakdown field strength of AOHBP/EPDM becomes lower than that of neat EPDM, indicating that the voltage stabilizer’s effect on improving the DC breakdown strength of EPDM diminishes. AOHBP graft-modification increases and decreases the breakdown voltage of EPDM by 2.7~13.5% and 3.7~37.4% under the pressures below and above 0.3 MPa as a critical turning point, respectively. This may be due to two reasons: First, under pressure, the average free volume inside the EPDM material decreases, partially suppressing the acceleration and destructive effects of high-energy electrons, making the role of voltage stabilizers less prominent. Second, the breakdown behavior of the material under mechanical stress may be related to localized weak points caused by stress, meaning factors such as structural uniformity, mechanical properties, intermolecular forces, and microscopic mechanical relaxation play a more significant role in this process. In this context, voltage stabilizers may have a negative impact.

In particular, when pressure rises up to 0.6 MPa, neat EPDM’s breakdown strength drops to 56.85 kV/mm (65.9% reduction from 0 MPa: 166.84 kV/mm), while AOHBP/EPDM exhibits 35.06 kV/mm (71.6% reduction). Each 0.1 MPa increase causes an average 10.9% reduction in the breakdown strength of neat EPDM, closely matching 11.2% reported by reference and validating the pressure-induced molecular chain defect mechanism. The observed voltage stabilizer efficacy turning negative above 0.3 MPa pressure in this study, a pressure-threshold effect absent in the literature, offers new insights for high-voltage cable accessory design.

### 3.5. Breakdown Characteristics of EPDM/XLPE Bilayer Dielectric Interface

**Figure 13:**
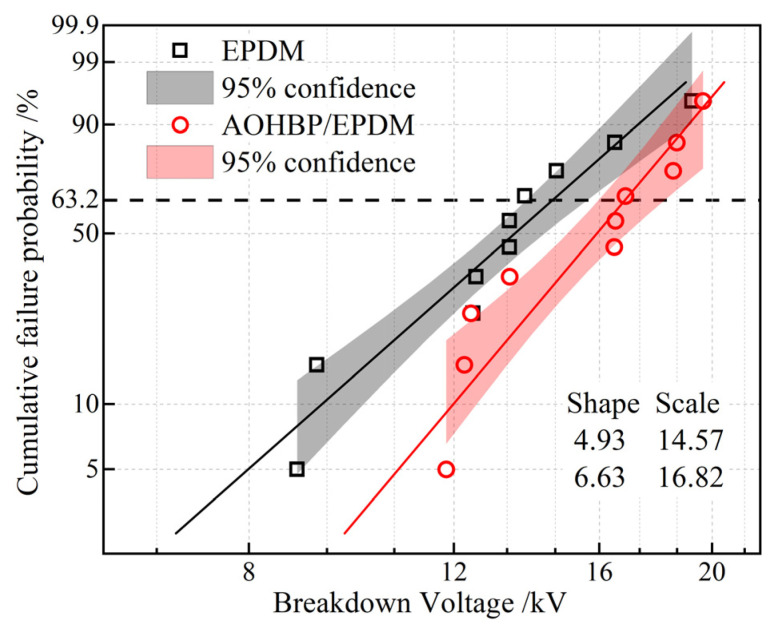
Weibull statistics on the breakdown voltages of EPDM-XLPE and AOHBP/EPDM-XLPE bilayers.

As indicated by the breakdown voltages of EPDM-XLPE and AOHBP/EPDM-XLPE bilayers (represent the composite dielectric interface) in Figure 13, it is verified that grafting AOHBP can increase the breakdown voltage by 2.25 kV at the interface between EPDM reinforced insulation and XLPE main insulation in cable accessory.

From the perspective of the insulating interface structure, theoretically, for an ideal defect-free insulating interface composed of two dielectrics, its dielectric strength inevitably depends on the electrical properties of the shallow surface layer of the material with lower bulk dielectric strength (EPDM). However, for a non-ideal insulating interface containing microvoid defects, the interface consists of microvoids wrapped by the two dielectrics and tightly connected points in series. The formation of a complete breakdown channel at this interface must pass through multiple tightly connected points and microvoids. The voltage stabilizer molecules can enhance the breakdown strength of the tightly connected points. Meanwhile, for microvoid defects filled with silicone grease, the voltage stabilizer-modified EPDM forms one of the inner surfaces of these microvoids. The voltage stabilizer facilitates the dissipation of surface charges and suppresses partial discharges within the microvoid defects through its charge trapping effect and excitation effect. As a result, the formation of a breakdown channel penetrating the interface is hindered, thereby improving the dielectric strength of the EPDM-XLPE interface.

For mitigating the reduction in DC breakdown field strength of EPDM under pressure which is a challenge in practical applications, we propose the following solutions and insights based on the findings of this study and the engineering needs of cable accessories:

(1) Based on the discovery in this study that 0.3 MPa critical pressure for the efficacy of grafting the voltage stabilizer, a gradient distribution of AOHBP is proposed: by controlling the crosslinking reaction rate, the surface layer of EPDM (0~50 μm) forms higher density shallow traps, while the inner layer maintains moderate crosslinking to preserve mechanical properties.
(2) Employing the synergistic effect of the “mechanical support” of nanoparticles and the “charge regulation” of voltage stabilizers by filling 1 wt% nanosilica combined with grafting 0.5 phr AOHBP to modify EPDM, in which nanosilica inhibits molecular chain slippage caused by pressure and hereby reduce the generation of microscopic defects, whilst AOHBP maintains shallow trap density to suppress charge accumulation.
(3) In response to possible pressure fluctuations during operation (such as thermal expansion and contraction caused by load changes), an “elastic buffer layer + pressure monitoring” scheme is suggested: 0.5 mm thick silicone rubber buffer layer is inserted at the interface between EPDM and XLPE to absorb pressure changes through its elastic deformation, and a micro pressure sensor is simultaneously implanted for monitoring interface pressure in real-time. When the pressure exceeds 0.3 MPa, this scheme can trigger a warning and adjust the load through temperature control to avoid long-term overpressure operation.

## 4. Limitations and Boundary Conditions

While certain achievements unraveling the modification effects of voltage stabilizer AOHBP on the insulation performance of EPDM cable accessories have been made, the inevitable limitations exist due to experimental conditions and research scope constraints, necessitating clear definition of the applicability conditions focused in the present research as follows:

(1) Experimental environment

All electrical performance tests (such as DC breakdown and surface flashover experiments) in this study were conducted under room temperature (25 ± 2 °C) and dry air conditions (relative humidity < 50%), without considering the impact of extreme temperatures (e.g., −40 °C low temperature or 80 °C high temperature), high humidity, or contaminated environments on the modification effects. Since actual cable accessories often operate in complex environments, temperature cycling may alter the migration characteristics of voltage stabilizers, while humidity may affect the dissipation paths of surface charges. Therefore, the applicability of this study’s conclusions under extreme environmental conditions requires further validation.

(2) Material system

It is focused solely on the modification effects of AOHBP as a voltage stabilizer on EPDM, which does not explore the modification differences among various types of voltage stabilizers (e.g., aromatic amines or hindered phenols) or consider the influence of parameters such as EPDM molecular weight and third monomer content on the modification effects. Additionally, the interfacial study of bilayer dielectrics only involves the EPDM/XLPE system, without investigating the interfacial characteristics between EPDM and other insulating materials (e.g., silicone rubber).

(3) Breakdown mechanism under pressure

Although the present study confirms the influence of pressure on the breakdown performance of EPDM through mechanical tests, it does not delve deeply into the coupling mechanism between pressure and electric fields (e.g., dynamic breakdown characteristics under alternating pressure) or establish a quantitative mathematical model linking pressure, trap evolution, and breakdown strength. Consequently, the conclusion regarding the “pressure threshold effect” is based solely on experimental observations, and the theoretical explanation requires further depth. In engineering applications, targeted validation should be conducted in conjunction with specific structural designs.

## 5. Conclusions

This study investigates the impact of graftable aromatic ketone voltage stabilizers on improving the surface flashover characteristics of EPDM. The addition of AOHBP reduces the energy level of EPDM and increases the density of shallow traps, thereby lowering the carrier transition energy and enhancing the surface flashover voltage of EPDM as well as the interfacial breakdown voltage between EPDM and XLPE. Under pressure, the DC breakdown field strength of EPDM and the effectiveness of the voltage stabilizer diminish as pressure increases. The breakdown behavior of EPDM under pressure may be related to changes in the material’s free volume and mechanical stress-induced damage.

In summary, AOHBP can modify EPDM through grafting reactions, significantly improving its surface flashover performance while optimizing crosslinking degree and inhibiting crystallization. This provides a theoretical foundation and technical reference for the performance optimization of EPDM materials used in cable accessories.

## Figures and Tables

**Table 1:** Characteristic crystallization parameters of EPDM and 0.5 phr AOHBP/EPDM.

Parameters	EPDM	AOHBP/EPDM
Melting Peak Temperature/°C	38.26	38.57
Crystallization Peak Temperature/°C	22.53	22.75

**Table 2:** DSC parameters in crosslinking reaction of EPDM and 0.5 phr AOHBP/EPDM.

Parameters	EPDM	AOHBP/EPDM
Exothermic peak temperature/°C	180.13	182.25
Enthalpy change of reaction Δ*H*/(J/g)	11.82	17.37

## Data Availability

The original contributions presented in this study are included in the article. Further inquiries can be directed to the corresponding author.
